# Association between Abdominal Obesity and Incident Colorectal Cancer: A Nationwide Cohort Study in Korea

**DOI:** 10.3390/cancers12061368

**Published:** 2020-05-26

**Authors:** Ga Eun Nam, Se-Jin Baek, Hong Bae Choi, Kyungdo Han, Jung-Myun Kwak, Jin Kim, Seon-Hahn Kim

**Affiliations:** 1 Department of Family Medicine, College of Medicine, Korea University, Seoul 02841, Korea; namgaaa@daum.net; 2 Department of Surgery, College of Medicine, Korea University, Seoul 02841, Korea; xezin@korea.ac.kr (S.-J.B.); cozypoppy@gmail.com (H.B.C.); jmkwak@korea.ac.kr (J.-M.K.); drkimsh@korea.ac.kr (S.-H.K.); 3 Department of Statistics and Actuarial Science, Soongsil University, Seoul 06978, Korea; hkd917@naver.com

**Keywords:** abdominal obesity, waist circumference, colorectal neoplasm, body mass index

## Abstract

Background: We investigated the association of w May aist circumference (WC) and abdominal obesity with the incident colorectal cancer risk in Korean adults. Methods: This nationwide population-based cohort study was based on health insurance claims data. We analyzed data from 9,959,605 participants acquired through health check-ups of the Korean National Health Insurance Service in 2009 who were followed up until the end of 2017. We performed multivariable Cox proportional hazards regression analysis. Results: During 8.3 years of follow up, 101,197 cases (1.0%) of colorectal cancer were recorded. After adjusting for potential confounders, there was a positive association between WC and colorectal cancer risk (*p* for trend <0.001). Abdominal obesity was associated with an increased risk of colorectal (hazard ratio: 1.10, (95% confidence interval: 1.08–1.12)), colon (1.11, 1.09–1.13), and rectal cancer (1.08, 1.05–1.10). These associations were independent of body mass index and were more pronounced in men and elderly individuals. Conclusion: We revealed that higher WC is related to colorectal cancer risk, thus suggesting that abdominal obesity may be a risk factor for colorectal cancer in this East Asian population.

## 1. Introduction

Globally, the prevalence of obesity has tripled over the past four decades with about 13% of the adult being obese worldwide in 2016 [1]. The obesity prevalence in Korea was 40.7% for men and 24.5% for women in 2015 [2]. Obesity is a serious and complex chronic disease requiring strategies for prevention and treatment and is associated with higher risks of various chronic diseases and mortality [3]. Obesity is also associated with the development of breast, endometrial, ovarian, colorectal, esophageal, renal, pancreatic, and prostatic cancers [4]. Some evidence has proposed that abdominal obesity may also increase the incidence of several cancers [5].

Colorectal cancer is viewed as an obesity-associated cancer with an ever-increasing incidence worldwide. It is the third most common and the second most common cancer globally and in Korea, respectively [6,7]. Prior studies suggested that lifestyle factors such as smoking, drinking, diet, physical activity, obesity, and environmental pollution influence the development of colorectal cancer. The colorectal cancer incidence has increased in Korea, possibly because of the obesity epidemic and Westernized diets [8]. Although mechanisms linking obesity and colorectal cancer remain unknown, changes in sex hormones, chronic inflammation, and genomic damage associated with obesity may affect colorectal cancer development [9]. Several studies showed that abdominal obesity is more frequently related with the occurrence of colorectal cancer among individuals with general obesity than among those without. This finding implies that abdominal obesity may play an additional role in the incidence of colorectal cancer compared to a simple increase in body mass index (BMI) [3,10], because abdominal obesity reflects body composition and metabolic health status considerably more than general obesity. This may be especially important in Asian populations because metabolically unhealthy normal weight is prevalent in such populations [11]. However, while the association between abdominal obesity and colorectal cancer has been largely studied in Western countries [12], evidence is limited regarding this association in Asian populations [8]. Therefore, we investigated the association of waist circumference (WC) and abdominal adiposity with incident colorectal cancer using nationwide data of South Korea.

## 2. Results

### 2.1. Baseline Characteristics

Table 1 shows the baseline characteristics of the participants (*n* = 9,959,605) regarding the development of colorectal cancer. Among all the participants, 101,197 (1.0%) developed colorectal cancer through the median follow-up duration of 8.3 years. The mean age and proportion of men were higher among individuals who developed colorectal cancer than among those who did not. The proportions of heavy alcohol drinkers and individuals with low income were higher in the incident colorectal cancer group compared to the group without colorectal cancer. According to our data, male sex, older age, great alcohol intake, and low incomes were more prevalent in individuals who developed colorectal cancer than those who did not. The mean BMI, WC, cardiometabolic parameters such as blood pressure, serum fasting glucose, lipid profiles except high-density lipoprotein cholesterol, and the proportions of hypertension, diabetes mellitus (DM), and dyslipidemia were higher among individuals who developed colorectal cancer than among those who did not.

### 2.2. Incidence and Risk of Colorectal, Colon, and Rectal Cancers by WC Categories

**Figure 1:**
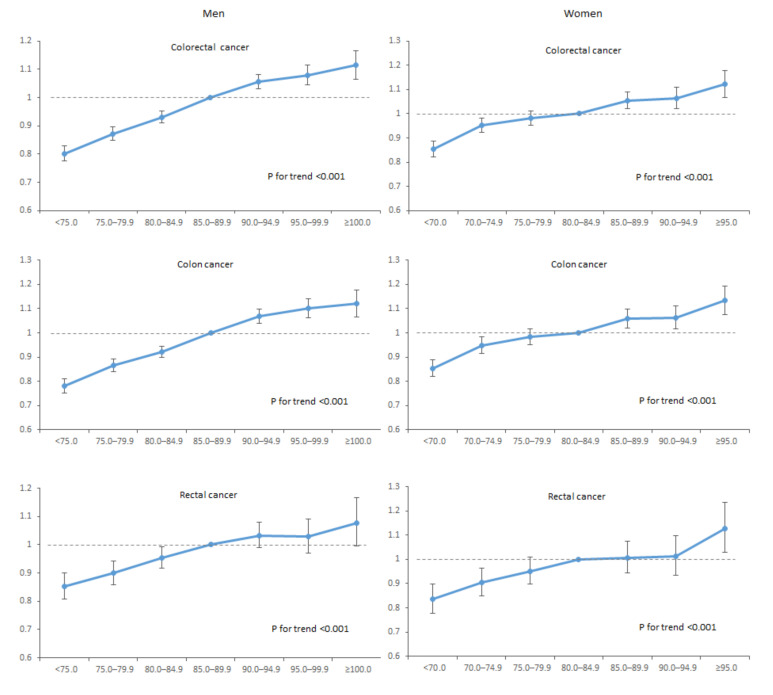
Associations between waist circumference and study outcomes among men and women.

Table 2 shows the longitudinal associations of WC categories with the incidence and risks of study outcomes. After adjusting for potential confounding variables, every 5 cm increase in WC was associated with 3.3%, 3.5%, and 3.0% higher risks of colorectal, colon, and rectal cancer (hazard ratio (HR) 1.033, 95% confidence interval (CI): 1.029–1.037 for colorectal cancer; 1.035, 1.031–1.040 for colon cancer; and 1.030, 1.023–1.038 for rectal cancer in model 2). These associations persisted after additionally adjusting for baseline BMI (model 3). For each 5 cm increment of WC, the HRs of incident colorectal, colon, and rectal cancer significantly increased in higher WC groups (*p* for trend <0.001). These associations were similarly observed for colon and rectal cancers, even after adjustment for baseline BMI (model 3) for all three outcomes. Individuals with abdominal obesity had a 10%, 11%, and 8% higher risk of colorectal, colon, and rectal cancer, respectively, compared with individuals without abdominal obesity in model 2 (HR 1.10, 95% CI: 1.08–1.12; 1.11, 1.09–1.13; and 1.08, 1.05–1.10, respectively); these associations were not considerably attenuated by further adjustment for baseline BMI. In addition, Figure 1 presents the longitudinal associations between WC categories and outcomes in men and women. After stratifying by sex, the positive associations between WC and colorectal, colon, and rectal cancers were consistently observed both in men and women (all *p* for trend <0.001).

### 2.3. Subgroup Analysis

**Figure 2:**
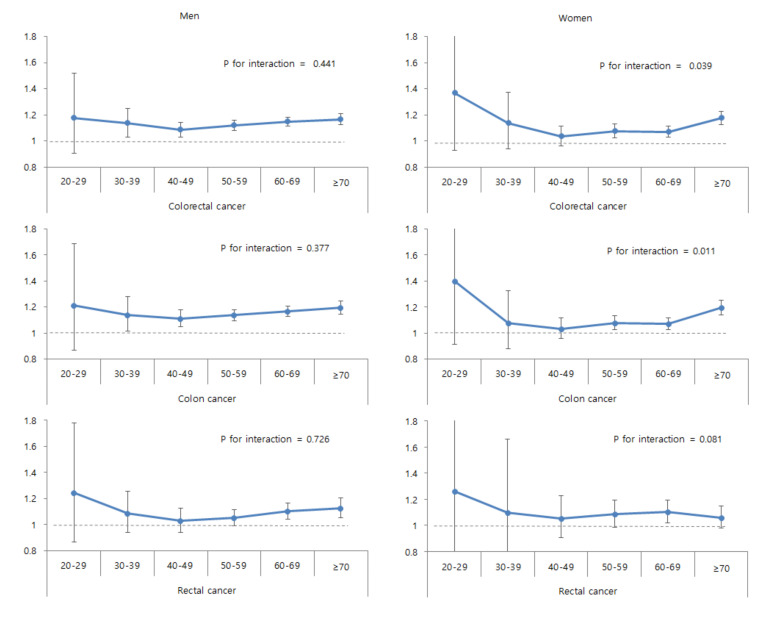
Associations between abdominal obesity and study outcomes according to age groups among men and women.

Associations of abdominal obesity with risks of study outcomes in subgroups are shown in Table 3. Abdominal obesity was strongly associated with incident colon cancer in elderly individuals (≥65 years) than in younger individuals (<65 years) (*p* for interaction = 0.024). The associations between abdominal obesity and colorectal, colon, and rectal cancers were more prominent among men than women. Similarly, such associations were more prominent in individuals without DM than in those with DM. The associations of abdominal obesity with colorectal and colon cancer were also more prominent in obese individuals (*p* for interactions = 0.037 and 0.016). Figure 2 shows the associations between abdominal obesity and outcomes according to age groups among men and women. While the associations between abdominal obesity and the risks of colorectal, colon, and rectal cancers were similar according to age groups among men (*p* for interaction >0.05), abdominal obesity was associated with colorectal and colon cancer risk more prominently in older age groups among women, especially in those ≥70 years of age) (*p* for interaction = 0.039 and 0.011).

### 2.4. Study Outcomes by the Combination of Obesity Status

Table 4 shows the adjusted HRs (95% CIs) of study outcomes by the combination of obesity status. Among the total participants, those with abdominal obesity alone and those with both types of obesity had increased HRs of the study outcomes compared to those in the group of participants without either type of obesity. However, general obesity alone was not associated with the risk of any outcomes (*p* < 0.001). Stratified analysis by sex revealed interactions between the associations and sex (*p* for interaction <0.001). In men, the HRs of the outcomes increased in participants with general obesity alone, abdominal obesity alone, and those with both types of obesity compared to those without any type of obesity (*p* < 0.001). The HRs of the study outcomes increased from those with abdominal obesity alone, general obesity alone, to those with both types in women (*p* < 0.001).

## 3. Discussion

From this cohort study of the Korean population, higher WC was associated with increased colorectal cancer risks, and abdominal obesity was associated with 10%, 11%, and 8% higher risks of colorectal, colon, and rectal cancers, respectively. These associations were continued after considering potential confounders, including baseline BMI. The associations were stronger in elderly individuals than in younger individuals, in men than in women, in individuals without DM than in those with DM, and in generally obese individuals than in individuals without general obesity. We found that higher WC is independently related with incident colorectal cancer and that abdominal obesity may be a factor related to colorectal cancer risk.

Prior studies examining the relation between obesity indices and incident colorectal cancer have produced conflicting results. Two recent meta-analyses reported a positive association, comparable to our findings. A meta-analysis from 53 prospective studies reported that higher BMI and WC levels were associated with increased colorectal cancer risk (relative risk, 95% CI: 1.334, 1.253–1.420 for BMI; 1.455, 1.327–1.596 for WC) [13]. Another meta-analysis from 18 cohort studies reported that larger WC and waist-to-hip ratio (WHR) were related with colorectal cancer occurrence (relative risk, 95% CI: 1.42, 1.30–1.55 for WC; 1.39, 1.25–1.53 for WHR) [14]. In addition, The International Agency for Research on Cancer (IARC) reported that the absence of overweight lowers the risk of colorectal cancer (relative risk of the highest BMI, 95% CI: 1.3, 1.3–1.4) [15]. In their prospective study, Fernando et al. observed that colonic polyps were frequently found during colonoscopies of individuals with abdominal obesity than others (50.2% vs. 22.8%, *p* = 0.03), and the risk increased with the severity of obesity (odds ratio 1.053, 95% CI: 1.034–1.071; *p* < 0.001) [16]. On the other hand, other studies have shown results conflicting with ours. A prospective cohort study of approximately 40,000 individuals found no associations between WC, weight change, and colorectal cancer [17]; however, the study was limited to middle-aged individuals from one city, unlike the present study, which included about 10 million adults nationwide of all ages.

The combination of lifestyle, environmental, and genetic factors contributes to cancer development. Obesity may increase cancer risk by affecting somatic mutations due to environmental factors [18]. The exact mechanism of the present results is unclear. However, several studies have proposed possible mechanisms related to obesity-related hormones. Obesity is related to increases in the levels of insulin, insulin-like growth factor (IGF), and adipocyte-derived factors such as tumor necrosis factor (TNF)-alpha, leptin, and interleukin (IL)-6 and a decrease in levels of adiponectin [19]. Insulin and the IGF system play key roles in colorectal cancer development, progression, and prognosis [20]. Obese individuals with greater abdominal obesity are more likely to have insulin resistance and hyperinsulinemic factors, which can further promote tumor growth [10]. Adiponectin as an insulin sensitizer and a negative regulator of angiogenesis mainly secreted from visceral adipose tissue, has been shown to inhibit colorectal cancer in animal experiments and has been related to colorectal cancer risk in clinical trials [19].

In this study, abdominal obesity was more closely correlated with colon cancer than with rectal cancer. A previous meta-analysis has reported that obesity is related with an increased risk of colorectal cancer at more variable rates than normal and that the relationship with obesity is stronger for colon cancer than for rectal cancer [21]. According to Dong et al., the relative risk for WC for rectal and colon cancer was 1.20 and 1.53, respectively [14]. The exact pathophysiology of the increased associated risk of abdominal obesity with colon cancer compared to that with rectal cancer is unknown but may be related to the anatomical differences between the colon and rectum. In rectal cancer, the mesorectum remains constant regardless of obesity status because the pelvis is a closed space, whereas the mesocolon thickens in obese patients with colon cancer. Therefore, it is difficult for the mesorectum to accurately reflect obesity; thus, its association with rectal cancer is relatively low.

In our study, abdominal obesity was more strongly associated with outcomes in men than in women. Previous studies reported similar trends to our findings. In a cohort study of Chinese participants, central obesity increased the colorectal cancer risk in men compared with that in women [22]. Larsson et al. also reported a positive relationship between obesity and colon and rectal cancer occurrence in both men and women, but especially in men [23]. The difference in cancer occurrence by sex is thought to be mainly related to hormonal differences between men and women. Fat is inversely related to testosterone levels in men but is positively correlated with estrogen levels in women [24]. A notable difference is developed in body composition between men and women where women have a larger percentage of body fat. In addition, the distribution of fat varies by sex, in which men have a relatively higher central distribution of fat. With weight gain, abdominal circumference is higher in men than in women, while women have more subcutaneous adipose tissue in the abdomen and thighs and lower visceral fat mass than men [25,26]. In general, abdominal obesity, represented by WC, indirectly reflects visceral fat, which is more associated with the onset of colorectal cancer than is subcutaneous fat [27,28]. In men, as opposed to women, abdominal obesity alone was more strongly related to colorectal cancer risk than general obesity in our study. This is because WC in women does not adequately reflect visceral fat due to their appreciable subcutaneous fat, while WC in men better reflects the amount of visceral fat. To address this problem, the use of abdominal CT to measure visceral fat can provide more accurate results, as reported recently [29,30]. 

This study has several limitations. First, the retrospective design prevented confirmation of causal relationships and there may be reverse causality despite our efforts regarding the washout period. Second, due to the free national screening, selection bias is possible as a well-screened person may have better health care. Third, although we used WC to assess abdominal obesity, WC might not accurately reflect the fat distribution. Fourth, we could not confirm the stage and exact anatomic location of colorectal cancers and presence or not of any metastases, because our study was based on the claim database from the Korean National Health Insurance System (NHIS). Fifth, we could not consider dietary habits such as meat consumption, which are related to both obesity and colorectal cancer risk. Nevertheless, our study is based on a database containing a large population of about 10 million people and has the strong advantage of being a well-researched study with adjustments for many potential confounders and subgroup analysis that suggested the association between abdominal obesity and colorectal cancer in East Asians.

## 4. Materials and Methods 

### 4.1. Study Setting and Population

We analyzed the health check-up data of the Korean National Health Insurance System (NHIS), managed by the Korean National Health Insurance Corporation (NHIC). The NHIS, as a single obligatory health insurance system, covers about 97% of the Korean population and provides, for all insured people, a health examination at least once every two years. Therefore, its database contains health information of approximately 50 million Koreans such as demographics, health check-ups, and disease diagnosis with its corresponding medical treatment based on the International Classification of Disease, 10th Revision, Clinical Modification (ICD-10-CM) codes. From this database, we initially considered 10,490,491 individuals aged ≥20 years who underwent health check-ups performed by the Korean NHIS between 1 January 2009 and 31 December 2009. We then excluded individuals with any missing variables (*n* = 417,814) and those diagnosed with colorectal cancer between 1 January 2002 and the time of enrollment (*n* = 113,072). Finally, we included 9,959,605 individuals (5,501,772 men and 4,457,833 women) and tracked them until 31 December 2017. The median duration of follow up was 8.3 (interquartile range: 8.1–8.6) years. This study adhered to the Declaration of Helsinki principles, and the institutional review board of Korea University Anam Hospital (number: 2019AN0151) approved the protocol. The requirement of informed consent was waived because the data are publicly open and de-identified.

### 4.2. Study Outcomes

The study endpoint was the occurrence of colorectal, colon, and rectal cancer from the index date to the end of 2017. The study outcomes were defined based on the ICD-10-CM codes (C18 or C19 for colon cancer; C20 for rectal cancer; and C18, C19, or C20 for colorectal cancer) and the registration code for cancer (V193). A copayment reduction registration program for intractable diseases has been conducted by the Korean government.

### 4.3. Categories of WC

Participants’ weight, height, and WC were measured. BMI was calculated as the weight (kg) divided by the height (m) squared. General obesity was defined as BMI ≥ 25 kg/m^2^ based on the World Health Organization definition of obesity for Asian populations [31]. Based on the WC cutoff for abdominal obesity in the Asian-specific population, we defined abdominal obesity as WC ≥90 cm in men and ≥85 cm in women [32]. We classified participants into seven groups at 5 cm WC intervals: <75.0, 75.0–79.9, 80.0–84.9, 85.0–89.9, 90.0–94.9, 95.0–99.9, ≥100.0 cm in men and <70.0, 70.0–74.9, 75.0–79.9, 80.0–84.9, 85.0–89.9, 90.0–94.9, and ≥95.0 cm in women.

### 4.4. Covariates 

Demographic and health behaviors were assessed using a standardized self-reported questionnaire. Income level was divided into quartiles. We defined individuals who smoked ≥100 cigarettes in their lifetime and who were smoking currently as current smokers. We defined individuals who drank ≥30 g/day of alcohol as heavy drinkers [33]. Individuals who performed strenuous exercise ≥3 times/week for ≥20 min or moderate exercise ≥5 times/week for ≥30 min were defined as regular exercisers. Blood pressures were measured after at least 5 min of rest. Serum fasting glucose level and lipid profile were measured after the participants fasted overnight. Hypertension was defined as blood pressure ≥140/90 mmHg or at least one claim for prescribing antihypertensive medications under ICD-10-CM codes I10–I13 or I15 per year. DM was defined as a fasting glucose level ≥126 mg/dL or at least one claim for prescribing antidiabetic medications in a given year under ICD-10-CM codes E11–E14. Dyslipidemia was defined as a serum total cholesterol level ≥240 mg/dL or at least one claim per year for prescribing lipid-lowering medications under the ICD-10-CM code E78.

### 4.5. Statistical Analysis

SAS version 9.4 (SAS Institute, Cary, NC, USA) was used for data analysis. We presented baseline characteristics as means ± standard deviation or numbers (percentage) and compared them using the independent t-test or Chi-square test. The incidence rates of the outcomes were calculated by dividing the number of events by 1000 person-years. We conducted multivariable Cox proportional hazard regression analysis to assess the association between WC categories and study outcomes; HRs and 95% CIs were calculated. Model 1 was unadjusted, while model 2 was adjusted for age, sex, smoking status, alcohol consumption, physical activity, income, hypertension, diabetes mellitus, and dyslipidemia. Model 3 was further adjusted for baseline BMI. We performed subgroup analyses and calculated *p*-values for the interactions between abdominal obesity and the subgroups for the study outcomes using a Cox regression analysis. We considered *p* < 0.05 statistically significant.

## 5. Conclusions

In conclusion, we found that higher WC is associated with incident colorectal cancer in Koreans and, independent of BMI, abdominal obesity itself affected colon cancer risk. This risk tended to be more pronounced in men and for colon cancer. Therefore, the prevention of colon cancer requires active control of abdominal obesity.

## Figures and Tables

**Table 1:** Baseline characteristics of the study population.

Characteristic	Colorectal Cancer		*p* ^1^
	No	Yes	
*n*	9,858,408	101,197	
Age (years)	46.8 ± 14.0	59.3 ± 11.7	<0.001
Sex (male)	5,440,501 (55.2)	61,271 (60.6)	<0.001
Current smoker	2,616,381 (26.5)	24,405 (24.1)	<0.001
Heavy alcohol drinker	790,083 (8.0)	9785 (9.7)	<0.001
Regular exerciser	1,812,993 (18.4)	21,304 (21.1)	<0.001
Income (lowest quartile)	2,584,903 (26.2)	27,341 (27.0)	<0.001
Height (cm)	164.0 ± 9.2	162.1 ± 8.7	<0.001
Weight (kg)	64.0 ± 11.6	63.5 ± 10.6	<0.001
Body mass index (kg/m^2^)	23.7 ± 3.2	24.1 ± 3.1	<0.001
Waist circumference (cm)	80.2 ± 9.1	83.0 ± 8.6	<0.001
Systolic blood pressure (mmHg)	122.4 ± 14.9	127.0 ± 15.9	<0.001
Diastolic blood pressure (mmHg)	76.3 ± 10.06	78.1 ± 10.2	<0.001
Glucose (mg/dL)	97.0 ± 22.8	103.3 ± 28.1	<0.001
Total cholesterol (mg/dL)	195.0 ± 36.6	197.7 ± 37.8	<0.001
Triglycerides ^2^	113.7 (113.7–113.8)	124.7 (124.3–125.1)	<0.001
HDL-C	55.7 ± 21.8	54.4 ± 24.4	<0.001
LDL-C	114.2 ± 47.0	115.7 ± 42.4	<0.001
Hypertension	2,498,816 (25.4)	44,630 (44.1)	<0.001
Diabetes mellitus	842,122 (8.5)	17,305 (17.1)	<0.001
Dyslipidemia	1,785,678 (18.1)	25,471 (25.2)	<0.001

^1^ Obtained using independent *t*-test or Chi-square test. ^2^ Values are presented as medians (interquartile range) using Wilcoxon rank-sum test.

**Table 2:** Incidence and risks of study outcomes according to waist circumference (WC) categories.

WC Category	*n*	Events	Person-Years	IR ^1^	HR (95% CI)		
					Model 1 ^2^	Model 2 ^3^	Model 3 ^4^
Colorectal cancer							
WC (per 5 cm)					1.18 (1.18–1.19)	1.033 (1.029–1.037)	1.040 (1.033–1.046)
WC (cm)							
M: <75.0, F: <70.0	1,724,651	9794	14,213,668	0.69	0.47 (0.46–0.48)	0.90 (0.88–0.92)	0.89 (0.86–0.91)
M: 75.0–79.9, F: 70.0–74.9	1,937,992	15,181	15,975,402	0.95	0.64 (0.63–0.66)	0.94 (0.92–0.96)	0.93 (0.91–0.95)
M: 80.0–84.9, F: 75.0–79.9	2,371,404	23,630	19,513,766	1.21	0.82 (0.80–0.83)	0.96 (0.94–0.98)	0.95 (0.94–0.97)
M: 85.0–89.9, F: 80.0–84.9	1,974,893	24,032	16,220,342	1.48	1 (ref.)	1 (ref.)	1 (ref.)
M: 90.0–94.9, F: 85.0–89.9	1,156,111	16,485	9,470,747	1.74	1.18 (1.15–1.20)	1.05 (1.03–1.07)	1.06 (1.04–1.08)
M: 95.0–99.9, F: 90.0–94.9	514,810	7797	4,208,225	1.85	1.25 (1.22–1.28)	1.06 (1.03–1.08)	1.06 (1.04–1.09)
M: ≥100.0, F: ≥95.0	279,744	4278	2,278,692	1.88	1.27 (1.23–1.31)	1.08 (1.05–1.12)	1.10 (1.06–1.14)
P					<0.001	<0.001	<0.001
*p* for trend					<0.001	<0.001	<0.001
M: <90.0, F: <85.0	8,008,940	72,637	65,923,178	1.10	1 (ref.)	1 (ref.)	1 (ref.)
M: ≥90.0, F: ≥85.0	1,950,665	28,560	15,957,664	1.79	1.62 (1.60–1.65)	1.10 (1.08–1.12)	1.08 (1.06–1.10)
Colon cancer							
WC (per 5 cm)					1.181 (1.177–1.186)	1.035 (1.031–1.040)	1.039 (1.032–1.046)
WC (cm)							
M: <75.0, F: <70.0	1,724,651	8114	14,213,668	0.57	0.46 (0.45–0.48)	0.90 (0.88–0.92)	0.90 (0.87–0.93)
M: 75.0–79.9, F: 70.0–74.9	1,937,992	12,621	15,975,402	0.79	0.64 (0.63–0.66)	0.94 (0.92–0.96)	0.94 (0.91–0.96)
M: 80.0–84.9, F: 75.0–79.9	2,371,404	19,543	19,513,766	1.00	0.81 (0.80–0.83)	0.96 (0.94–0.97)	0.96 (0.94–0.97)
M: 85.0–89.9, F: 80.0–84.9	1,974,893	20,011	16,220,342	1.23	1 (ref.)	1 (ref.)	1 (ref.)
M: 90.0–94.9, F: 85.0–89.9	1,156,111	13,890	9,470,747	1.47	1.19 (1.16–1.22)	1.06 (1.04–1.09)	1.06 (1.04–1.09)
M: 95.0–99.9, F: 90.0–94.9	514,810	6600	4,208,225	1.57	1.27 (1.24–1.31)	1.07 (1.04–1.10)	1.07 (1.04–1.10)
M: ≥100.0, F: ≥95.0	279,744	3624	2,278,692	1.59	1.29 (1.25–1.34)	1.09 (1.05–1.13)	1.09 (1.05–1.14)
P					<0.001	<0.001	<0.001
*p* for trend					<0.001	<0.001	<0.001
M: <90.0, F: <85.0	8,008,940	60,289	65,923,178	0.91	1 (ref.)	1 (ref.)	1 (ref.)
M: ≥90.0, F: ≥85.0	1,950,665	24,114	15,957,664	1.51	1.65 (1.63–1.68)	1.11 (1.09–1.13)	1.08 (1.06–1.10)
Rectal cancer							
WC (per 5 cm)					1.196 (1.189–1.203)	1.030 (1.023–1.038)	1.049 (1.037–1.061)
WC (cm)							
M: <75.0, F: <70.0	1,724,651	2945	14,213,668	0.21	0.46 (0.44–0.48)	0.89 (0.85–0.93)	0.84 (0.80–0.88)
M: 75.0–79.9, F: 70.0–74.9	1,937,992	4547	15,975,402	0.28	0.62 (0.60–0.65)	0.92 (0.88–0.95)	0.89 (0.85–0.92)
M: 80.0–84.9, F: 75.0–79.9	2,371,404	7342	19,513,766	0.38	0.83 (0.80–0.85)	0.96 (0.93–0.99)	0.94 (0.91–0.98)
M: 85.0–89.9, F: 80.0–84.9	1,974,893	7397	16,220,342	0.46	1 (ref.)	1 (ref.)	1 (ref.)
M: 90.0–94.9, F: 85.0–89.9	1,156,111	4914	9,470,747	0.52	1.14 (1.10–1.18)	1.02 (0.99–1.06)	1.04 (1.002–1.08)
M: 95.0–99.9, F: 90.0–94.9	514,810	2259	4,208,225	0.54	1.18 (1.12–1.23)	1.02 (0.97–1.06)	1.05 (1.00–1.10)
M: ≥100.0, F: ≥95.0	279,744	1266	2,278,692	0.56	1.22 (1.15–1.29)	1.08 (1.02–1.15)	1.15 (1.08–1.23)
P					<0.001	<0.001	<0.001
*p* for trend					<0.001	<0.001	<0.001
M: <90.0, F: <85.0	8,008,940	22,231	65,923,178	0.34	1 (ref.)	1 (ref.)	1 (ref.)
M: ≥90.0, F: ≥85.0	1,950,665	8439	15,957,664	0.53	1.57 (1.53–1.61)	1.08 (1.05–1.10)	1.07 (1.04–1.10)

Abbreviations: IR, incidence rate; HR, hazard ratio; CI, confidence interval; WC, waist circumference; M, male; F, female. ^1^ Incidence per 1000 person-years. ^2^ Model 1 was unadjusted. ^3^ Model 2 was adjusted for age, sex, smoking status, alcohol consumption, physical activity, income, hypertension, diabetes mellitus, and dyslipidemia. ^4^ Model 3 was adjusted for age, sex, smoking status, alcohol consumption, physical activity, income, hypertension, diabetes mellitus, dyslipidemia, and body mass index.

**Table 3:** Subgroup analysis.

Subgroup	Colorectal Cancer	*p* for Interaction	Colon Cancer	*p* for Interaction	Rectal Cancer	*p* for Interaction
Age (years)		0.102		0.024		0.959
<65	1.07 (1.05–1.09)		1.08 (1.05–1.10)		1.05 (1.02–1.09)	
≥65	1.15 (1.13–1.18)		1.18 (1.15–1.20)		1.11 (1.06–1.15)	
Sex		<0.001		<0.001		0.003
Male	1.15 (1.13–1.17)		1.17 (1.15–1.20)		1.09 (1.06–1.13)	
Female	1.11 (1.08–1.13)		1.11 (1.08–1.14)		1.09 (1.04–1.14)	
Diabetes mellitus		0.003		0.003		0.041
No	1.11 (1.09–1.12)		1.12 (1.10–1.14)		1.09 (1.05–1.12)	
Yes	1.08 (1.05–1.11)		1.09 (1.05–1.13)		1.04 (0.99–1.10)	
Body mass index (kg/m^2^)		0.037		0.016		0.738
<25	1.07 (1.04–1.10)		1.07 (1.04–1.10)		1.07 (1.02–1.12)	
≥25	1.10 (1.07–1.12)		1.10 (1.08–1.13)		1.08 (1.03–1.12)	
Smoking		0.331		0.340		0.444
Non-smoker	1.12 (1.10–1.14)		1.13 (1.11–1.15)		1.09 (1.06–1.12)	
Current smoker	1.09 (1.05–1.12)		1.09 (1.06–1.13)		1.08 (1.03–1.14)	

Hazard ratio (95% confidence interval) was calculated using multivariable Cox regression analysis after adjusting for age, sex, smoking status, alcohol consumption, physical activity, income, hypertension, diabetes mellitus, and dyslipidemia.

**Table 4:** Risk of study outcomes according to the combined presence of general obesity and abdominal obesity.

Sex	General Obesity	Abdominal Obesity	HR (95% CI)		
			Colorectal Cancer	Colon Cancer	Rectal Cancer
Total	No	No	1 (ref.)	1 (ref.)	1 (ref.)
	No	Yes	1.06 (1.03–1.09)	1.06 (1.03–1.10)	1.08 (1.02–1.13)
	Yes	No	1.01 (0.99–1.03)	1.01 (0.99–1.03)	1.02 (0.98–1.05)
	Yes	Yes	1.11 (1.10–1.13)	1.13 (1.11–1.15)	1.08 (1.05–1.11)
P			<0.001	<0.001	<0.001
Men	No	No	1 (ref.)	1 (ref.)	1 (ref.)
	No	Yes	1.14 (1.10–1.17)	1.14 (1.10–1.19)	1.15 (1.09–1.22)
	Yes	No	1.05 (1.03–1.08)	1.06 (1.03–1.09)	1.04 (1.00–1.08)
	Yes	Yes	1.17 (1.15–1.19)	1.20 (1.18–1.23)	1.09 (1.05–1.13)
P			<0.001	<0.001	<0.001
Women	No	No	1 (ref.)	1 (ref.)	1 (ref.)
	No	Yes	1.04 (1.00–1.08)	1.04 (1.00–1.09)	0.99 (0.91–1.08)
	Yes	No	1.05 (1.02–1.08)	1.06 (1.03–1.10)	1.02 (0.96–1.08)
	Yes	Yes	1.14 (1.11–1.17)	1.15 (1.12–1.18)	1.13 (1.07–1.18)
P			<0.001	<0.001	<0.001
*p* for interaction			<0.001	<0.001	<0.001

Hazard ratio (95% confidence interval) was calculated using multivariable Cox regression analysis after adjusting for age, sex, smoking status, alcohol consumption, physical activity, income, hypertension, diabetes mellitus, and dyslipidemia.
